# Auranofin, an Anti-rheumatic Gold Drug, Aggravates the Radiation-Induced Acute Intestinal Injury in Mice

**DOI:** 10.3389/fphar.2019.00417

**Published:** 2019-04-24

**Authors:** Eun Sang Lee, Joong Sun Kim, Hyounji Lee, Jee-Yeon Ryu, Hae-June Lee, Jong Kyung Sonn, Young-Bin Lim

**Affiliations:** ^1^Division of Radiation Biomedical Research, Korea Institute of Radiological and Medical Sciences, Seoul, South Korea; ^2^Herbal Medicine Resources Research Center, Korea Institute of Oriental Medicine, Daejeon, South Korea; ^3^Department of Biology, College of Natural Sciences, Kyungpook National University, Daegu, South Korea

**Keywords:** auranofin, intestinal mucosa, apoptosis, oxidative stress, thioredoxin reductase, radiosensitivity

## Abstract

Pelvic and abdominal radiotherapy plays an important role in eradication of malignant cells; however, it also results in slight intestinal injury. The apoptosis of cells in the intestinal epithelium is a primary pathological factor that initiates radiation-induced intestinal injury. Auranofin, a gold-containing triethylphosphine, was approved for the treatment of rheumatoid arthritis, and its therapeutic application has been expanded to a number of other diseases, such as parasitic infections, neurodegenerative disorders, AIDS, and bacterial infections. Recently, a treatment strategy combining the use of auranofin and ionizing radiation aimed at increasing the radiosensitivity of cancer cells was proposed for improving the control of local cancers. In this study, we evaluated the effect of auranofin on the radiosensitivity of intestinal epithelial cells. The treatment with a combination of 1 μM auranofin and 5 Gy ionizing radiation showed clear additive effects on caspase 3 cleavage and apoptotic DNA fragmentation in IEC-6 cells, and auranofin administration significantly aggravated the radiation-induced intestinal injury in mice. Auranofin treatment also resulted in the activation of the unfolded protein response and in the inhibition of thioredoxin reductase, which is a key component of the cellular antioxidant system. Pre-treatment with N-acetyl cysteine, a well-known scavenger of reactive oxygen species, but not with a chemical chaperone, which inhibits endoplasmic reticulum stress and the ensuing unfolded protein response, significantly reduced the radiosensitizing effects of auranofin in the IEC-6 cells. In addition, transfection of IEC-6 cells with a small interfering RNA targeted against thioredoxin reductase significantly enhanced the radiosensitivity of these cells. These results suggest that auranofin-induced radiosensitization of intestinal epithelial cells is mediated through oxidative stress caused by the deregulation of thioredoxin redox system, and auranofin treatment can be an independent risk factor for the development of acute pelvic radiation disease.

## Introduction

Gold (atomic number 79) is a metal belonging to Group IB in the periodic table. The therapeutic value of gold compounds has been known since ancient times. In the modern era, the use of gold compounds in medicine was initiated with the detection of anti-tubercular activity of gold(I) dicyanide by Robert Koch (Kean et al., 1997). This observation led investigators to experiment with the use of gold compounds for treating various diseases including rheumatoid disease. Controlled clinical trials proved the efficacy of gold compounds in the treatment of rheumatoid arthritis in 1960 (The Empire Rhematism Council, 1960). Auranofin, a gold-containing triethylphosphine, serves as reserve therapy for rheumatoid arthritis. Although, it was originally approved for the treatment of rheumatoid arthritis in 1985, its therapeutic application has been expanded to a number of other diseases, such as parasitic infections, neurodegenerative disorders, AIDS, and bacterial infections (Roder and Thomson, 2015). Recently, auranofin was approved by US Food and Drug Administration for phase II clinical trials for cancer therapy^1^. The mechanism of therapeutic action of auranofin involves the inhibition of TrxR, which is a key component of the cellular antioxidant system, and induction of endoplasmic reticulum (ER) stress and subsequent activation of the UPR (Fan et al., 2014; Fiskus et al., 2014; Liu et al., 2014).

Radiotherapy is a proven principal therapeutic tool in the treatment of various tumors; however, damage to radiosensitive tissue and cell types, with high rates of cell division, limits its use (Sinclair and Morton, 1966; Little et al., 1973). For example, pelvic and abdominal radiotherapy can generate intestinal injury, causing acute pelvic radiation disease. About half to three-fourth of patients undergoing pelvic and abdominal radiotherapy develop acute symptoms of pelvic radiation disease, such as diarrhea, abdominal pain, nausea, and vomiting (Shadad, 2013; Morris and Haboubi, 2015). The burden of pelvic radiation disease-related symptoms, which affect the quality of the life of a patient, has been under-appreciated and sub-optimally managed. Currently, management of the pelvic toxicities can range from anti-inflammatory treatment to reconstructive surgery depending on severity (Cosentino and Piro, 2018; Matta et al., 2019). A number of patient-related factors, such as inflammatory bowel disease, diabetes, and collagen vascular disease, are strongly correlated with the incidence and severity of the pelvic radiation disease (Chon and Loeffler, 2002). Apoptosis in the intestinal epithelium is the primary pathological factor that initiates the radiation-induced intestinal injury (Potten et al., 1994; MacNaughton, 2000; Ch’ang et al., 2005; Burdelya et al., 2008). Recently, a treatment strategy combining the use of auranofin and ionizing radiation (IR) was proposed to increase the radiosensitivity of cancer cells with the aim of improving the local control of cancer (Wang et al., 2017). It remains unclear, however, whether auranofin has the potential to affect the incidence and severity of IR-induced damage to surrounding non-cancerous tissues, especially to radiosensitive tissues. The aim of the present study was to determine whether auranofin treatment is an independent factor that influences the risk of intestinal complications after pelvic or abdominal radiotherapy. For this purpose, we evaluated the effects of auranofin treatment on radiation-induced apoptosis, which is the primary pathological factor that initiates the radiation-induced injury, in the intestinal epithelium.

## Materials and Methods

### Cell Culture

The IEC-6 cell line was purchased from American Type Culture Collection (Manassas, VA, United States) and cultured in Dulbecco’s Modified Eagle Medium (DMEM) with 10% fetal bovine serum at 37°C under an atmosphere of 5% CO_2_. This cell line was developed from normal rat intestinal crypt cells (Ametani et al., 1996). The experiments were performed using the cells passaged for a maximum of 10 times.

### Materials and Irradiation

Auranofin and tunicamycin were purchased from Sigma-Aldrich (St Louis, MO, United States). TUDCA was purchased from TCI America (Portland, OR, United States). Irradiation was performed at room temperature using a ^137^Cs gamma-ray source, Gammacell 3000, manufactured by Nordion (Ottawa, ON, Canada).

### Quantitative Reverse Transcription-Polymerase Chain Reaction (qRT-PCR)

Total RNA was isolated from cells using a Hybrid-R Kit (GeneAll Biotechnology, Seoul, South Korea). RNA (1 μg) was subjected to reverse transcription with PrimeScript RT master mix, according to the manufacturer’s protocol (Takara, Tokyo, Japan). PCR was performed in triplicates using a CFX96^TM^ Real-Time system (Bio-Rad, Hercules, CA, United States) and qPCR SYBR Green master mix (m.biotech, Gyeonggi, South Korea). The primers for qRT-PCR were designed using AmplifX program^2^ and were purchased from Integrated DNA Technologies (Coralville, IA, United States). The relative expression levels of each targeted gene were normalized to the average expression of β-actin (F-TCCTTCCTGGGTATGGAATCCTGT and R-CTCCTTCTGCATCCTGTCAGCAAT) and GAPDH (F-ACGACCCCTTCATTGACCTCAACT and R-GATGGTGATGGGTTTCCCGTTGAT) by using the ΔΔC_T_ comparative method (Livak and Schmittgen, 2001). The following transcripts were measured using the indicated forward and reverse primers: thioredoxin reductase 1 (Txnrd1): F-TGTGGATGCTGTTGCCAAGACT and R-AACCCAAGAGCCTTCCTGAACT; thioredoxin reductase 2 (Txnrd2): F-GCAAATGGCGTCTTTGGTCACA and R-TGCAGTTGGTTAGTCGGGAGTT; X-box-binding protein 1s (Xbp1s): F-CTGAGTCCGAATCAGGTGCAG and ATCCATGGGAAGATGTTCTGG; 78 KDa glucose-regulated protein (grp78): F-TGGGTACATTTGATCTGACTGGA and R-CTCAAAGGTGACTTCAATCTGGG.

### Western Blotting

Total cell lysates were prepared using RIPA buffer, and western blot analyses were performed as described previously (Lim et al., 2000). The anti-cleaved CASPASE 3 (1:2000) antibody was purchased from Cell Signaling Technology (Cat# number 9661; Danvers, MA, United States) and anti-β ACTIN (1:2000) was purchased from Sigma-Aldrich (Cat# A1978; St Louis, MO, United States).

### Small Interfering RNA and Cell Transfection

Small interfering RNA oligonucleotides were designed using the siRNA-designing tool provided by Dharmacon Research (Lafayette, CO, United States). The oligonucleotide sequences were as follows: 5′-GGGCAUCCCUGGAGACAAA-dTdT-3′ (*Txnrd1* siRNA), 5′-CAGCACAACUGGAAGGCAA-dTdT-3′ (*Txnrd*2 siRNA), and 5′-AAAUGAACGUGAAUUGCUCAA-dTdT-3′ (luciferase siRNA). Transfection was performed using the RNAiMAX protocol provided by Invitrogen (Carlsbad, CA, United States). After 24 h, the transfected cells were used for experiments.

### Thioredoxin Reductase Activity Assay

The activity of TrxR was measured using TrxR colorimetric assay kit, according to the manufacturer’s protocol (Cayman Chemical, Ann Arbor, MI, United States). Briefly, the cell lysate was incubated with a colorimetric substrate, 5,5′-dithiobis-2-nitrobenzoate, which was reduced to produce yellow colored 5-thio-2-nitrobenzoic acid (TNB). The TrxR-catalyzed formation of TNB was measured by reading the absorbance at 405 nm.

### Determination of Intracellular Hydrogen Peroxide Concentration

The intracellular concentration of H_2_O_2_ was determined using hydrogen peroxide assay kit, according to the manufacturer’s protocol (Abcam, Cambridge, MA, United States). Briefly, deproteinized cell lysate was reacted with OxiRed probe and horse radish peroxidase (HRP) in a 96-well microplate wrapped in a foil at room temperature. The HRP-catalyzed release of a red-fluorescing oxidation product was quantitated by measuring the absorbance at 405 nm.

### Animals and Irradiation

Six-week-old female C3H mice were purchased from Central Lab (Animal Inc., Seoul, South Korea), and the experiments were performed after 1 week of quarantine and acclimatization. All the protocols used in this study were approved by the Institutional Animal Care and Use Committee of the Korean Institute of Radiological and Medical Sciences (IACUC permit number: KIRAMS217-0007). Each mouse was anesthetized with tiletamine/zolazepam (Zoletil 50^®^, Virak Korea, Seoul, South Korea) and exposed to 10 and 13 Gy of IR using an X-RAD 320 system (Precision X-ray, North Branford, CT, United States) at 250 kV, 10 mA with 420 mm aluminum added filtration at a dose rate of 2 Gy/min. The sham-irradiated mice were treated in exactly the same manner as the irradiated animals but were not irradiated. For quantitation of apoptosis, the mice were sacrificed after 12 h of IR (10 Gy) exposure, and their small intestines were fixed in 10% neutral-buffered formaldehyde and embedded in paraplast wax to prepare 4-μm-thick sections. Apoptosis in the cells present in paraffin sections was detected by TUNEL immunostaining, and the TUNEL-positive cells in the paraffin sections containing a large portion of the crypt base, the lumen, and at least 17 cells along the crypt column were counted under an optical microscope. Two investigators (Joong Sun Kim and Eun Sang Lee) performed the immunohistochemistry study, and were blinded in terms of the treatment groups.

### Jejunal Crypt Assay and Morphological Changes

For histological examination, mice were sacrificed 3.5 days after exposure to 13 Gy IR. Their small intestines were fixed in 10% neutral-buffered formaldehyde and embedded in paraplast wax to prepare 4-μm-thick tissue sections of the jejunum for hematoxylin and eosin (H&E) staining. Duplicate sections of four different parts of the jejunum from each animal were prepared for histological examination. The regenerating crypts observed in the cross-sections of the jejunum were then counted. To analyze the morphological changes, all the samples were sectioned and arranged into successive slices to search for the sample with the longest villi. This sample was used because it yielded more homogenous results than those of standard techniques based merely on the measurement of the 10 longest villi for a single slice of the sample. The images of intestinal sections were obtained with a digital camera mounted on a Nikon Eclipse 80i microscope (Nikon Corporation). The quantification was performed using Image-Pro Plus image analysis software (Media Cybernetics, Bethesda, United States).

### Statistical Analysis

SPSS 23.0 software (SPSS, Inc., Chicago, IL, United States) was used for all the statistical analyses. The results were expressed as the mean ± standard deviation (SD) values of three experiments that were performed independently. The normal distribution of variables was assessed using the Kolmogorov–Smirnov test. Statistical analyses were performed using the Student’s *t*-test or ANOVA with Bonferroni *post hoc* test for multiple comparisons, and *P*-values less than 0.05 were considered statistically significant.

## Results

### Auranofin Enhanced the IR-Induced Cell Death in IEC-6 Cells

**FIGURE 1:**
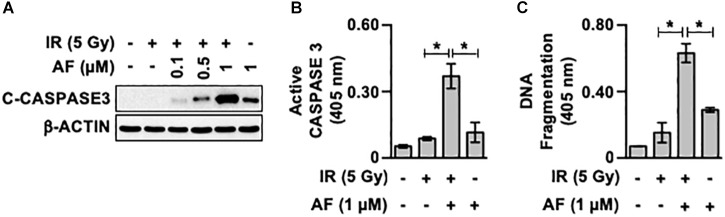
Auranofin radiosensitizes the IEC-6 cells. **(A)** IEC-6 cells were pretreated with or without the indicated concentrations of auranofin (AF) for 1 h and were subsequently treated with or without 5 Gy of ionizing radiation (IR) for 16 h. The cleavage of caspase 3 (C-caspase3) was then examined by western blotting. **(B)** IEC-6 cells were pretreated with or without 1 μM AF for 1 h and were subsequently treated with or without 5 Gy of IR for 16 h. The caspase 3 activity was then determined using the CaspACE Assay System (Promega). **(C)** IEC-6 cells were pretreated with or without 1 μM AF for 1 h and were subsequently treated with or without 5 Gy IR for 24 h. The internucleosomal DNA fragmentation was then determined using the Cell Death Detection ELISA^PLUS^ kit (Roche). Results were replicated in three independent experiments (mean values ± SD; Student’s *t*-test; ^∗^, *P* < 0.05).

To determine whether auranofin affects the radiosensitivity of intestinal epithelial cells, we assessed the changes in the activity of caspase 3 in the IR-exposed IEC-6 cells in response to auranofin treatment. Interestingly, 1 μM of auranofin treatment could trigger mild cleavage of caspase 3 in the IEC-6 cells, however, a significant increase in the degree of auranofin-induced caspase 3 cleavage was observed when it was used in combination with IR (Figure 1A). The co-treatment with auranofin and IR also showed obvious additive effects on the caspase 3 activity (Figure 1B) and apoptotic DNA fragmentation (Figure 1C). These data indicate that auranofin enhances the radiosensitivity of IEC-6 cells.

### Auranofin Induced ER Stress Precedes Caspase 3 Activation

**FIGURE 2:**
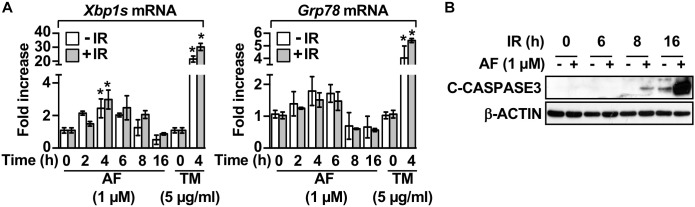
Auranofin activates the unfolded protein response in the IEC-6 cells. **(A)** IEC-6 cells were pretreated for 1 h with 1 μM AF or 5 μg/mL tunicamycin (TM) and were subsequently treated with or without 5 Gy IR. Relative amounts of *Xbp1s* and *Grp78* mRNAs were quantified by real-time PCR at the indicated time points after treatment with 5 Gy IR in the IEC-6 cells. **(B)** IEC-6 cells were pretreated for 1 h with or without 1 μM AF. The cleavage of caspase 3 was then determined by western blotting at the indicated time points after treatment with 5 Gy IR in the IEC-6 cells. Results were replicated in three independent experiments (mean values ± SD; two-way ANOVA with Bonferroni *post hoc* test; ^∗^, *P* < 0.05).

We examined whether the mechanisms underlying auranofin-mediated radiosensitization of IEC-6 cells involved the UPR pathway by measuring the auranofin-induced alterations in this pathway. The treatment of IEC-6 cells with auranofin induced the UPR pathway, as evidenced by the enhanced expression of *Xbp1s* and *Grp78* mRNAs, but no further increase in the degree of induction of the UPR pathway by auranofin was observed when it was used in combination with 5 Gy IR (Figure 2A). Tunicamycin, an inhibitor of protein glycosylation, was used as a positive control for UPR induction (Figure 2A,B). As shown in Figure 2A, a significant increase in the levels of *Xbp1s* and *Grp78* mRNAs was evident within 4 h of IR treatment in the IEC-6 cells pretreated with auranofin, but an increase in caspase 3 cleavage in the IEC-6 cells pretreated with auranofin was evident at 16 h after IR treatment (Figure 2B). Therefore, induction of the UPR pathway preceded caspase 3 activation, suggesting that the UPR signaling could contribute to the up-regulation of radiosensitivity in response to auranofin treatment in the IEC-6 cells.

### Chemical Chaperone Failed to Ameliorate the Auranofin-Induced Radiosensitization

**FIGURE 3:**
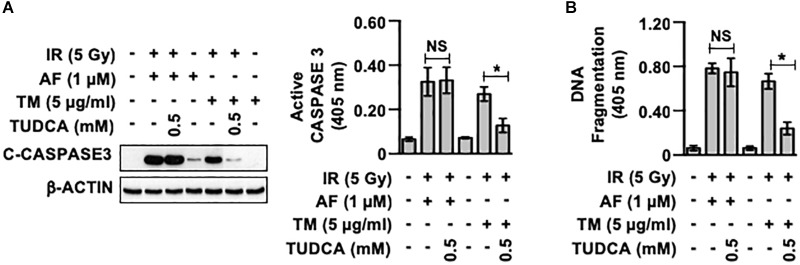
Tauroursodeoxycholic acid inhibits the radiosensensitization of IEC-6 cells induced by tunicamycin, but not by auranofin. **(A)** IEC-6 cells were pretreated for 1 h with or without 0.5 mM tauroursodeoxycholic acid (TUDCA) and subsequently treated with either 1 μM auranofin (AF) or 5 μg/ml tunicamycin (TM) for another 1 h. The cleavage of caspase 3 was then determined by western blotting (left panel), and caspase 3 activity was measured using the CaspACE Assay System (right panel) at 16 h after exposure of the IEC-6 cells to 5 Gy IR. **(B)** IEC-6 cells were pretreated for 1 h with or without 0.5 mM TUDCA and subsequently treated with either 1 μM AF or 5 μg/mL TM for another 1 h. The internucleosomal DNA fragmentation was then determined using the Cell Death Detection ELISA^PLUS^ kit at 24 h after exposure of the IEC-6 cells to 5 Gy IR. Results were replicated in three independent experiments (mean values ± SD; two-way ANOVA with Bonferroni *post hoc* test; ^∗^, *P* < 0.05).

To investigate whether activation of the UPR pathway observed in the IEC-6 cells treated with auranofin was involved in determining the radiosensitivity of these cells, we examined the effects of an ER stress inhibitor on the toxicity of IR to IEC-6 cells pretreated with auranofin by determining the caspase 3 cleavage. As shown in Figure 3A, treatment with TUDCA, a representative ER stress inhibitor (Lee et al., 2014), failed to block the induction of caspase 3 cleavage when auranofin was used in combination with IR. On the other hand, despite the higher activation of the UPR pathway in IEC-6 cells treated with tunicamycin as compared to that in cells treated with auranofin (Figure 2A), TUDCA treatment significantly inhibited the induction of caspase 3 cleavage observed when tunicamycin was used in combination with IR. In addition, TUDCA significantly inhibited the up-regulation of apoptotic DNA fragmentation in IEC-6 cells caused by co-treatment with tunicamycin and IR, but no alterations in the degree of IR-induced apoptotic DNA fragmentation in IEC-6 cells treated with auranofin were observed (Figure 3B). These data suggest that the UPR pathway is not involved in the regulation of radiosensitivity in response to auranofin treatment in IEC-6 cells.

### TrxR Activity Is Inhibited by Auranofin and Is Followed by an Increase in the H_2_O_2_ Concentration

**FIGURE 4:**
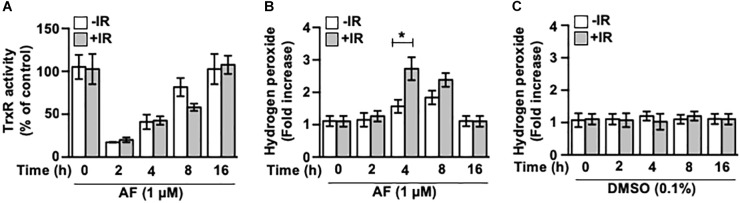
Auranofin inhibits the thioredoxin reductase (TrxR) activity in the IEC-6 cells. **(A)** IEC-6 cells were treated with or without 5 Gy IR at 1 h after 1 μM of auranofin treatment. The activity of total intracellular TrxR was measured using the thioredoxin reductase assay kit (Cayman) at the indicated time points after treatment of the IEC-6 cells with 1 μM of auranofin. **(B)** IEC-6 cells were treated with or without 5 Gy IR at 1 h after 1 μM of auranofin treatment. The level of intracellular hydrogen peroxide was determined using the hydrogen peroxide assay kit (abcam) at the indicated time points after treatment of IEC-6 cells with 1 μM of auranofin. **(C)** IEC-6 cells were treated with or without 5 Gy IR at 1 h after 0.1% DMSO treatment. The level of intracellular hydrogen peroxide was determined using the hydrogen peroxide assay kit (abcam) at the indicated time points after treatment of IEC-6 cells with 0.1% DMSO. Results were replicated in three independent experiments (mean values ± SD; Student’s *t*-test; ^∗^, *P* < 0.05).

We determined the effects of auranofin treatment on the TrxR activity and on the level of reactive oxygen species (ROS) in the IEC-6 cells. Within 2 h of auranofin treatment, the total intracellular TrxR activity decreased by >80%, but recovered thereafter (Figure 4A). No alterations in the activity of TrxR were observed after the IR treatment (data not shown), and the potent inhibitory effect of auranofin on TrxR activity was not affected by the IR exposure (Figure 4A). As shown in Figure 4B, auranofin treatment increased the level of H_2_O_2_ in the cells. However, exposure to 5 Gy IR alone led to no significant alterations in the level of H_2_O_2_ in the IEC-6 cells (Figure 4C), whereas an increased level of H_2_O_2_ was apparent in the presence of auranofin, and it was remarkably higher than that in cells treated with auranofin alone. This strengthening effect exerted by auranofin and IR on the H_2_O_2_ levels suggests that the measured level of H_2_O_2_ in the cells treated with auranofin and IR does not depend on the increase in its production, but rather on the decrease in its removal because of the inhibition of TrxR.

### TrxR System Regulates the Radiosensitivity of IEC-6 Cells by Determining the Cellular Antioxidant Capacity

**FIGURE 5:**
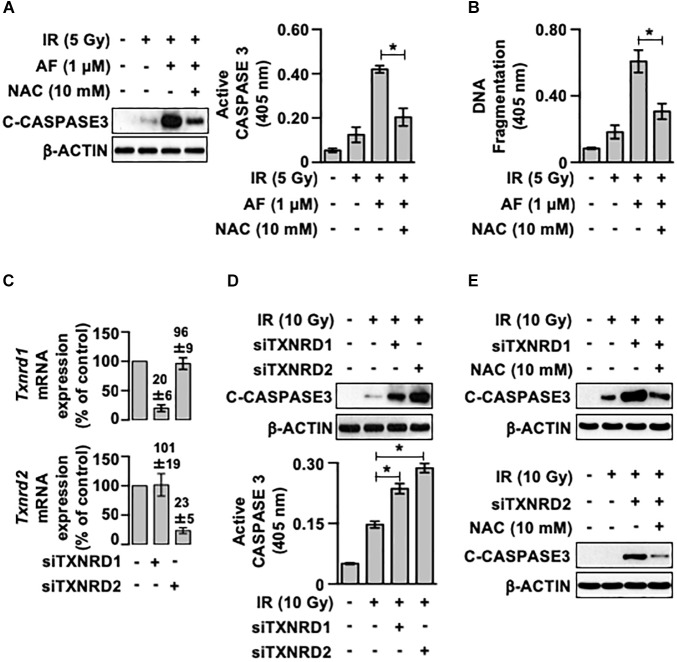
A scavenger of reactive oxygen species ameliorates the auranofin-induced radiosensitization of IEC-6 cells. **(A)** IEC-6 cells were pretreated for 1 h with or without 10 mM *N*-acetyl-L-cysteine (NAC) and subsequently treated with or without 1 μM auranofin (AF) for another 1 h. The cleavage of caspase 3 was then determined using western blotting (left panel), and the caspase 3 activity was measured using the CaspACE Assay System (right panel) at 16 h after exposure of the IEC-6 cells with 5 Gy IR treatment. **(B)** IEC-6 cells were pretreated for 1 h with or without 10 mM NAC and subsequently treated with or without 1 μM AF for another 1 h. The internucleosomal DNA fragmentation was then measured using the Cell Death Detection ELISA^PLUS^ kit at 24 h after the exposure of IEC-6 cells to 5 Gy IR treatment. **(C)** 24 h after transfection with scrambled siRNA, *thioredoxin reductase 1* siRNA (siTXNRD1), or *thioredoxin reductase 2* siRNA (siTXNRD2), the relative amounts of *Txnrd1* (upper panel) and *Txnrd2* (lower panel) mRNAs in the IEC-6 cells were quantified by real-time PCR. The RNA levels were normalized to those of *Gapdh* and scrambled siRNA-transfected controls. **(D)** 24 h after transfection with siTXNRD1 or siTXNRD2, the cleavage of caspase 3 was determined by western blotting at 16 h after the exposure of IEC-6 cells (upper panel) to 10 Gy IR treatment, and caspase 3 activity was measured using the CaspACE Assay System (lower panel). **(E)** 24 h after transfection with siTXNRD1 (upper panel) or siTXNRD2 (lower panel), the IEC-6 cells were pretreated with or without 10 mM NAC for 1 h. The cleavage of caspase 3 was then determined by western blotting at 16 h after exposure of the IEC-6 cells to 10 Gy IR treatment. Results were replicated in three independent experiments (mean values ± SD; two-way ANOVA with Bonferroni *post hoc* test; ^∗^, *P* < 0.05).

To determine whether auranofin-induced inhibition of TrxR and subsequent induction of oxidative stress contributed to the auranofin-induced radiosensitization of IEC-6 cells, we examined the effects of NAC, a well-known ROS scavenger, on the radiosensitivity of IEC-6 cells pretreated with auranofin. As shown in Figure 5A, pretreatment with NAC led to an inhibition in the induction of the up-regulation of caspase 3 activity observed when auranofin was used in combination with IR. Furthermore, NAC treatment significantly ameliorated the up-regulation of DNA fragmentation in the IEC-6 cells caused by co-treatment with auranofin and IR (Figure 5B). These observations suggest that the TrxR system might be a critical regulator of the radiosensitivity of IEC-6 cells. To confirm this hypothesis, we utilized siRNAs targeted against *Txnrd1* and *Txnrd2* transcripts (Figure 5C). As shown in Figure 5D, *Txnrd1* or *Txnrd2* knockdown enhanced the IR-induced caspase 3 activation. In addition, the enhanced caspase 3 activation caused by the *Txnrd* knockdown was significantly inhibited by NAC treatment (Figure 5E). These findings suggest that TrxR system is a critical regulator of the radiosensitivity of IEC-6 cells and it does so by regulating the antioxidant capacity of the cells, and the inhibition of TrxR system contributes to auranofin-induced radiosensitization of IEC-6 cells.

### Auranofin Aggravates the IR-Induced Acute Intestinal Injury

**FIGURE 6:**
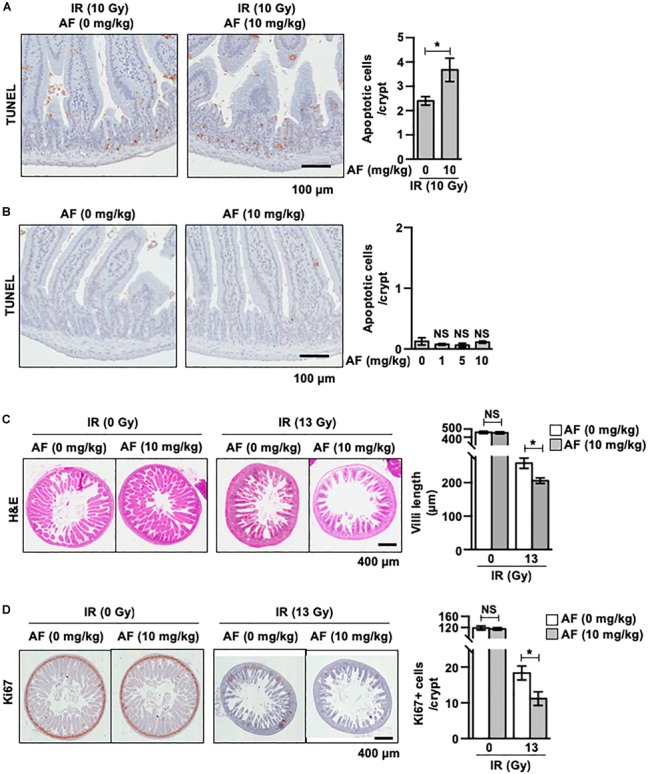
Non-toxic dose of auranofin aggravates ionic radiation-induced acute intestinal injury. **(A)** Auranofin (10 mg/kg) or saline solutions were given to C3H mice orally 5 times with 24 h interval before the exposure of IR. The apoptotic cells were identified by microscopic detection of the TUNEL-positive signal in histological sections of the proximal jejunal crypts harvested at 12 h after exposure to 10 Gy of IR, as described in Materials and Methods. The number of TUNEL-positive cells counted in 80 crypt sections, pooled from four mice, is presented as a histogram (mean values ± SD; Student’s *t*-test; ^∗^, *P* < 0.05). **(B)** Auranofin (0, 1, 5, and 10 mg/kg) was given to C3H mice orally 5 times with a 24 h interval before mock-irradiation. The apoptotic cells were identified by microscopic detection of TUNEL-positive signals in the histological section of proximal jejunal crypts harvested at 12 h after the mock-irradiation. The number of TUNEL-positive cells counted in 80 crypt sections, pooled from four mice, is presented as a histogram (mean values ± SD; Student’s *t*-test; ^∗^, *P* < 0.05). **(C)** Representative hematoxylin and eosin (H&E)-stained sections of jejunum harvested at 3.5 days after mock or 13 Gy irradiation. The length of villi was measured as described in Materials and Methods. The length measured in 80 crypt sections, pooled from four mice, is presented as a histogram (mean values ± SD; Student’s *t*-test; ^∗^, *P* < 0.05). **(D)** Representative Ki67 stained sections of jejunum harvested at 3.5 days after mock or 13 Gy irradiation. The number of Ki67+ cells counted in 80 crypt sections, pooled from four mice, is presented as a histogram (mean values ± SD; Student’s *t*-test; ^∗^, *P* < 0.05).

We used a mice model to evaluate the *in vivo* effect of using radiation and auranofin in combination. The quantification of apoptotic cell death in the crypts using TUNEL staining revealed the presence of apoptotic cells throughout the crypts at 12 h of exposure to 10 Gy of the subtotal IR (Figure 6A). The rate of IR-induced apoptotic cell death was 2.40 ± 0.18 in the crypts. The mice injected with auranofin showed more TUNEL positive cells in response to 10 Gy of the subtotal IR (Figure 6A), whereas the rate of apoptotic cell death observed in the group treated with auranofin alone was similar to that in the control mice (Figure 6B).

We next measured the effects of auranofin on the morphology of jejunum samples collected 3.5 days after exposure to subtotal IR at 13 Gy. As shown in Figure 6C, the height of the villi was significantly decreased in mice, 3.5 days after the IR exposure compared to that in the sham control group, demonstrating the radiation-induced injuries to the jejunal mucosa (Figure 6C). The radiation-induced injuries were aggravated by the administration of auranofin as evidenced by reduction in the length of villi (Figure 6C). The immunohistochemical detection of Ki-67 was performed to detect the proliferation of crypt cells. Consistent with the results obtained for the length of villi, the administration of auranofin aggravated the radiation-induced decrease in the proliferation of crypt cells (Figure 6D).

## Discussion

Apoptosis in the intestinal epithelium is the primary pathological factor that initiates radiation-induced intestinal injury. A dose of radiation of about 10 Gy sterilizes a large proportion of the dividing cells in the intestinal epithelium. In several mouse strains, the radiation doses lower than 14 Gy in a single dose causes sublethal intestinal injury, but there is no instance on record of a human having survived a dose in excess 10 Gy (Hall and Giaccia, 2012).

The lumen of ER provides a unique cellular environment for the folding and maturation of newly synthesized proteins (Araki and Nagata, 2012). A range of physiological and pathological insults, such as viral infections, sustained protein demands, and inflammatory cytokines, result in the accumulation of misfolded and unfolded proteins within the ER, a condition termed as ER stress (Fonseca et al., 2011; Hetz, 2012; Wang and Kaufman, 2012). Metazoans have evolved a conserved signaling cascade, referred to as the UPR, to protect against ER stress. The UPR has three branches requiring inositol-requiring enzyme 1 (IRE1), protein kinase RNA-like ER kinase (PERK), or activating transcription factor (ATF6). In response to ER stress, IRE1, PERK, and ATF6 initiate signal transduction processes to promote the expression of genes that are involved in the mitigation of ER stress (Fonseca et al., 2011; Hetz, 2012; Wang and Kaufman, 2012). However, when ER stress is prolonged or is excessive, these UPR signaling pathways lead to apoptotic death of the stressed cells.

The mechanisms of action of auranofin include the induction of ER stress and subsequently of the UPR. For example, auranofin induces lethal oxidative stress, which triggers ER stress and is followed by UPR in the primary chronic lymphocytic leukemia cells (Fiskus et al., 2014). SK053, a peptidomimetic compound targeting the TrxR system, induces apoptosis in tumor cells accompanied by the activation of the ER stress, and UPR regulates the SK053-induced apoptosis in tumor cells (Muchowicz et al., 2015). In addition, inactivation of peroxiredoxin 4, which comprises the TrxR system, by piperlongumine treatment was reported to exacerbate the intracellular ER stress, and the UPR pathway mediated the piperlongumine-induced apoptosis in glioma cells (Zou et al., 2015). In this study, auranofin treatment evoked acute UPR within 4 h in IEC-6 cells, and significantly enhanced the IR-induced apoptosis in these cells. Interestingly, this radiosensitizing effect of auranofin was not blocked by the pretreatment with an ER stress inhibitor, whereas the ER stress inhibitor completely inhibited the induction of caspase 3 cleavage observed when tunicamycin was used in combination with IR, suggesting that the UPR pathway evoked by auranofin does not contribute to the radiosensitizing effect of auranofin in IEC-6 cells.

TrxR, together with Trx and NADPH, comprise the Trx system, which is a key antioxidant system in defense against oxidative stress (Holmgren and Lu, 2010; Lu and Holmgren, 2014). Three TrxRs are present in mammalian cells, namely the cytosolic TrxR1, mitochondrial TrxR2, and a testis-specific thioredoxin glutathione reductase. The TrxRs provide electrons from NADPH to Trx, thereby, contributing to the antioxidant defense, both by catalyzing antioxidant reactions and by regulating other antioxidant enzymes, such as peroxiredoxin and methionine sulfoxide reductases (Holmgren and Lu, 2010; Lu and Holmgren, 2014). Mammalian TrxRs contain a selenocysteine residue in their active sites that is critical to their catalytic activity. Auranofin is known to inhibit the TrxRs by forming a stable adduct with the selenocysteine residue, resulting in the alteration in redox balance in various cells, which can lead to increased production of H_2_O_2_ that causes cellular oxidative stress (Saccoccia et al., 2014). In this study, treatment with auranofin resulted in an immediate inhibition of the TrxR activity, which was followed by an increase in the level of H_2_O_2_ that mediated the radiosensitizing effect of auranofin in the IEC-6 cells. Interestingly, no significant alterations in the intracellular concentration of H_2_O_2_ were observed in the IEC-6 cells treated with 5 Gy IR, but the irradiation caused a significant increase in the intracellular H_2_O_2_ concentration in auranofin treated IEC-6 cells as compared to that in the cells treated with auranofin alone, suggesting that the TrxR system actively mitigated the IR-induced oxidative stress. In support of this hypothesis, we observed that the knockdown of *Txnrd1* or *Txnrd2* enhanced the IR-induced caspase 3 activation, and the enhanced caspase 3 activation caused by Txnrd knockdown was significantly inhibited by treatment with NAC. Therefore, the TrxR system is a critical regulator of the radiosensitivity of intestinal epithelial cells and does so by regulating the cellular antioxidant capacity.

Abdominal pain, loose stools, and diarrhea are major adverse effects that limit the use of auranofin for the treatment of rheumatoid arthritis (van Riel et al., 1983; Behrens et al., 1986). The development of loose stools occurs in 40% of the patients taking auranofin. This symptom develops during the first few weeks of treatment and disappears promptly upon termination of the treatment. In addition, the auranofin treatment was reported to cause ulcerative colitis during the treatment of rheumatoid, which was accompanied by mucosal inflammation and crypt abscesses (Langer et al., 1987). However, it is unclear whether auranofin has the potential to affect the incidence and severity of radiation-induced intestinal injury.

## Conclusion

In this study, we found that a non-toxic dose of auranofin significantly aggravated the severity of the radiation-induced intestinal injury. This suggests that auranofin treatment can be an independent factor that influences the risk of intestinal complications after pelvic or abdominal radiotherapy.

## Ethics Statement

All the protocols used in this study were approved by the Institutional Animal Care and Use Committee of the Korean Institute of Radiological and Medical Sciences (IACUC permit number: KIRAMS217-0007).

## Author Contributions

H-JL, JS, and Y-BL designed the experiments. EL and JK conducted the experiments and performed the data analysis. HL and J-YR contributed in manuscrpript revision. All authors discussed the results and contributed to manuscript writing.

## Conflict of Interest Statement

The authors declare that the research was conducted in the absence of any commercial or financial relationships that could be construed as a potential conflict of interest.

## Footnotes

1 http://clinicaltrials.gov/ct2/show/NCT01419691

2 http://crn2m.univ-mrs.fr

## Abbreviations

NAC: *N*-acetyl-L-cysteine
siRNA: small interfering RNA
TrxR: thioredoxin reductase
TUDCA: tauroursodeoxycholic acid
TUNEL: terminal deoxynucleotidyl transferase-mediated dUTP-biotin nick-end labeling
UPR: unfolded protein response

## References

[B1] Ametani A. Hachimura S. Yamamoto Y. Shimizu M. Imaoka A. Yi H. K. (1996). Consecutive events of growth, differentiation and death of the small intestinal epithelial cell line, IEC-6. *In Vitro Cell. Dev. Biol. Anim.* 32 127–130. 10.1007/bf02723676 8925133

[B2] Araki K. Nagata K. (2012). Protein folding and quality control in the ER. *Cold Spring Harb. Perspect. Biol.* 4:a015438. 10.1101/cshperspect.a015438 22855729 PMC3405861

[B3] Behrens R. Devereaux M. Hazleman B. Szaz K. Calvin J. Neale G. (1986). Investigation of auranofin-induced diarrhoea. *Gut* 27 59–65. 10.1136/gut.27.1.59 3081411 PMC1433187

[B4] Burdelya L. G. Krivokrysenko V. I. Tallant T. C. Strom E. Gleiberman A. S. Gupta D. (2008). An agonist of toll-like receptor 5 has radioprotective activity in mouse and primate models. *Science* 320 226–230. 10.1126/science.1154986 18403709 PMC4322935

[B5] Ch’ang H.-J. Maj J. G. Paris F. Xing H. R. Zhang J. Truman J.-P. (2005). ATM regulates target switching to escalating doses of radiation in the intestines. *Nat. Med.* 11 484–490. 10.1038/nm1237 15864314

[B6] Chon B. H. Loeffler J. S. (2002). The effect of nonmalignant systemic disease on tolerance to radiation therapy. *Oncologist* 7 136–143. 10.1634/theoncologist.7-2-136 11961197

[B7] Cosentino D. Piro F. (2018). Hyaluronic acid for treatment of the radiation therapy side effects: a systematic review. *Eur. Rev. Med. Pharmacol. Sci.* 22 7562–7572. 10.26355/eurrev_201811_16298 30468506

[B8] Fan C. Zheng W. Fu X. Li X. Wong Y.-S. Chen T. (2014). Enhancement of auranofin-induced lung cancer cell apoptosis by selenocystine, a natural inhibitor of TrxR1 in vitro and in vivo. *Cell Death Dis.* 5:e1191. 10.1038/cddis.2014.132 24763048 PMC4001298

[B9] Fiskus W. Saba N. Shen M. Ghias M. Liu J. Gupta S. D. (2014). Auranofin induces lethal oxidative and endoplasmic reticulum stress and exerts potent preclinical activity against chronic lymphocytic leukemia. *Cancer Res.* 74 2520–2532. 10.1158/0008-5472.CAN-13-2033 24599128 PMC4172421

[B10] Fonseca S. G. Gromada J. Urano F. (2011). Endoplasmic reticulum stress and pancreatic β-cell death. *Trends Endocrinol. Metab.* 22 266–274. 10.1016/j.tem.2011.02.008 21458293 PMC3130122

[B11] Hall E. J. Giaccia A. J. (2012). *Radiobiology for the Radiologist.* Philadelphia, PA: Lippincott Williams & Wilkins.

[B12] Hetz C. (2012). The unfolded protein response: controlling cell fate decisions under ER stress and beyond. *Nat. Rev. Mol. Cell Biol.* 13 89–102. 10.1038/nrm3270 22251901

[B13] Holmgren A. Lu J. (2010). Thioredoxin and thioredoxin reductase: current research with special reference to human disease. *Biochem. Biophys. Res. Commun.* 396 120–124. 10.1016/j.bbrc.2010.03.083 20494123

[B14] Kean W. F. Hart L. Buchanan W. W. (1997). Auranofin. *Br. J. Rheumatol.* 36 560–572. 9189058 10.1093/rheumatology/36.5.560

[B15] Langer H. E. Hartmann G. Heinemann G. Richter K. (1987). Gold colitis induced by auranofin treatment of rheumatoid arthritis: case report and review of the literature. *Ann. Rheum. Dis.* 46 787–792. 10.1136/ard.46.10.787 3318725 PMC1003390

[B16] Lee E. S. Lee H.-J. Lee Y.-J. Jeong J.-H. Kang S. Lim Y.-B. (2014). Chemical chaperones reduce ionizing radiation-induced endoplasmic reticulum stress and cell death in IEC-6 cells. *Biochem. Biophys. Res. Commun.* 450 1005–1009. 10.1016/j.bbrc.2014.06.091 24973711

[B17] Lim Y. B. Kang S. S. Park T. K. Lee Y. S. Chun J. S. Sonn J. K. (2000). Disruption of actin cytoskeleton induces chondrogenesis of mesenchymal cells by activating protein kinase C-alpha signaling. *Biochem. Biophys. Res. Commun.* 273 609–613. 10.1006/bbrc.2000.2987 10873653

[B18] Little J. B. Hahn G. M. Frindel E. Tubiana M. (1973). Repair of potentially lethal radiation damage in vitro and in vivo. *Radiology* 106 689–694. 10.1148/106.3.689 4684814

[B19] Liu N. Li X. Huang H. Zhao C. Liao S. Yang C. (2014). Clinically used antirheumatic agent auranofin is a proteasomal deubiquitinase inhibitor and inhibits tumor growth. *Oncotarget* 5 5453–5471. 24977961 10.18632/oncotarget.2113 PMC4170648

[B20] Livak K. J. Schmittgen T. D. (2001). Analysis of relative gene expression data using real-time quantitative PCR and the 2(-Delta Delta C(T)) Method. *Methods* 25 402–408. 10.1006/meth.2001.1262 11846609

[B21] Lu J. Holmgren A. (2014). The thioredoxin antioxidant system. *Free Radic. Biol. Med.* 66 75–87. 10.1016/j.freeradbiomed.2013.07.036 23899494

[B22] MacNaughton W. K. (2000). Review article: new insights into the pathogenesis of radiation-induced intestinal dysfunction. *Aliment. Pharmacol. Ther.* 14 523–528. 10.1046/j.1365-2036.2000.00745.x 10792113

[B23] Matta R. Chapple C. R. Fisch M. Heidenreich A. Herschorn S. Kodama R. T. (2019). Pelvic complications after prostate cancer radiation therapy and their management: an international collaborative narrative review. *Eur. Urol.* 75 464–476. 10.1016/j.eururo.2018.12.003 30573316

[B24] Morris K. A. Haboubi N. Y. (2015). Pelvic radiation therapy: between delight and disaster. *World J. Gastrointest. Surg.* 7 279–288. 10.4240/wjgs.v7.i11.279 26649150 PMC4663381

[B25] Muchowicz A. Firczuk M. Wachowska M. Kujawa M. Jankowska-Steifer E. Gabrysiak M. (2015). SK053 triggers tumor cells apoptosis by oxidative stress-mediated endoplasmic reticulum stress. *Biochem. Pharmacol.* 93 418–427. 10.1016/j.bcp.2014.12.019 25573101

[B26] Potten C. S. Merritt A. Hickman J. Hall P. Faranda A. (1994). Characterization of radiation-induced apoptosis in the small intestine and its biological implications. *Int. J. Radiat. Biol.* 65 71–78. 10.1080/09553009414550101 7905913

[B27] Roder C. Thomson M. J. (2015). Auranofin: repurposing an old drug for a golden new age. *Drugs R D* 15 13–20. 10.1007/s40268-015-0083-y 25698589 PMC4359176

[B28] Saccoccia F. Angelucci F. Boumis G. Carotti D. Desiato G. Miele A. E. (2014). Thioredoxin reductase and its inhibitors. *Curr. Protein Pept. Sci.* 15 621–646. 10.2174/1389203715666140530091910 24875642 PMC4275836

[B29] Shadad A. K. (2013). Gastrointestinal radiation injury: prevention and treatment. *World J. Gastroenterol.* 19 199–208. 10.3748/wjg.v19.i2.199 23345942 PMC3547575

[B30] Sinclair W. K. Morton R. A. (1966). X-ray sensitivity during the cell generation cycle of cultured Chinese hamster cells. *Radiat. Res.* 29 450–474. 10.2307/3572025 5924188

[B31] The Empire Rhematism Council (1960). Gold therapy in rheumatoid arthritis. Report of a multicentre control trial. *Ann. Rheum. Dis.* 19 95–119. 10.1136/ard.19.2.95 13856636 PMC1007132

[B32] van Riel P. L. Gribnau F. W. van de Putte L. B. Yap S. H. (1983). Loose stools during auranofin treatment: clinical study and some pathogenetic possibilities. *J. Rheumatol.* 10 222–226. 6408256

[B33] Wang H. Bouzakoura S. de Mey S. Jiang H. Law K. Dufait I. (2017). Auranofin radiosensitizes tumor cells through targeting thioredoxin reductase and resulting overproduction of reactive oxygen species. *Oncotarget* 8 35728–35742. 10.18632/oncotarget.16113 28415723 PMC5482612

[B34] Wang S. Kaufman R. J. (2012). The impact of the unfolded protein response on human disease. *J. Cell Biol.* 197 857–867. 10.1083/jcb.201110131 22733998 PMC3384412

[B35] Zou P. Chen M. Ji J. Chen W. Chen X. Ying S. (2015). Auranofin induces apoptosis by ROS-mediated ER stress and mitochondrial dysfunction and displayed synergistic lethality with piperlongumine in gastric cancer. *Oncotarget* 6 36505–36521. 10.18632/oncotarget.5364 26431378 PMC4742192

